# Design of a Single-Cell Positioning Controller Using Electroosmotic Flow and Image Processing

**DOI:** 10.3390/s130506793

**Published:** 2013-05-21

**Authors:** Chyung Ay, Chao-Wang Young, Jhong-Yin Chen

**Affiliations:** Department of Biomechatronic Engineering, National Chiayi University, No.300, Shyefu Rd., East Dist., Chiayi 600, Taiwan; E-Mails: cay@mail.ncyu.edu.tw (C.A.); s1010363@mail.ncyu.edu.tw (J.-Y.C.)

**Keywords:** image processing, electroosmotic flow, fuzzy logic, automatic control, cell position

## Abstract

The objective of the current research was not only to provide a fast and automatic positioning platform for single cells, but also improved biomolecular manipulation techniques. In this study, an automatic platform for cell positioning using electroosmotic flow and image processing technology was designed. The platform was developed using a PCI image acquisition interface card for capturing images from a microscope and then transferring them to a computer using human-machine interface software. This software was designed by the Laboratory Virtual Instrument Engineering Workbench, a graphical language for finding cell positions and viewing the driving trace, and the fuzzy logic method for controlling the voltage or time of an electric field. After experiments on real human leukemic cells (U-937), the success of the cell positioning rate achieved by controlling the voltage factor reaches 100% within 5 s. A greater precision is obtained when controlling the time factor, whereby the success rate reaches 100% within 28 s. Advantages in both high speed and high precision are attained if these two voltage and time control methods are combined. The control speed with the combined method is about 5.18 times greater than that achieved by the time method, and the control precision with the combined method is more than five times greater than that achieved by the voltage method.

## 1. Introduction

The microscope has made a tremendous contribution to 21st century scientific research. In particular, the electron microscope, phase-contrast microscope and scanning tunneling microscope have provided improved clarity for the observation of biological specimens, furthering understanding at the atomic and molecular levels. Continuous, strong developments in nanotechnology have permitted the manipulation of microparticle movements. Developing microparticle manipulation techniques mainly utilizes a dielectrophoresis method which applies a high-frequency, and periodically high-intensity, electric field to enable regular movements, or movements for a single particle in a predetermined path within a microchannel. An electroosmotic phenomenon method that drives a one-dimensional movement is also utilized for microparticles contained in microfluids. For example, Oddy and Santiago analyzed the movement rate of particles subject to dielectrophoresis and electroosmosis in a microchannel under AC and DC electric fields [1]. Although these studies on microparticle manipulation techniques have yielded significant results, information concerning the automation of a series of operation and control steps is scarce. Many aspects of automation remain to be developed.

As early as the beginning of the 19th-century, Reuss discovered the electrokinetic force [2]; two hundred years later, Wang *et al.* introduced the idea that mixing within microchannels is an important process since certain microfluidic applications require the rapid mixing of species [3]. Their paper investigates mixing within microchannels using the simulation of a two-dimensional electrokinetically-driven flow where the microchannel is populated with patterned blocks. Pohl introduced dielectrophoresis to both biological and chemical fields in an effort to resolve the problem where uncharged particles fail to be driven by dielectrophoresis [4].

Presently, many experiments focus on single cells, and require their fixation. The researcher only selects the most special cell as sample in many cells, so the development of a nondestructive and automatic single-cell localization technique is necessary. Cell fixation methods include the use of the chemical agent Cell-Tak or optical tweezers to move a cell for fixation. Although both methods can fulfill the fixation purpose, the use of a chemical agent could affect the cell and optical tweezers could cause cell death within 200 s. Armani *et al.* showed how to combine microfluidics and feedback control to independently steer multiple particles with micrometer accuracy in two spatial dimensions [5]. The particles are steered by creating a fluid flow that carries all particles from where they are to where they should be at each time step. Therefore, cell lysis directly occurring on a chip can simplify the pre-treatment procedures for biotechnological analysis of samples.

Many studies for applications in recognition and control have been conducted. However, very few studies concerning applications in biomolecules on a two-dimensional plane, and automatic control for the free movement of a single cell have been undertaken. When processing and studying a single cell, automatic means requiring less time to achieve higher accuracy in the free control and localization of cells than would be realised through manual adjustment. The current study aims to use image processing to track a single cell [6], apply the electroosmotic flow to drive the movement of the cell, and employ fuzzy logic to provide control rules [7,8]. The purpose is to utilize a simple instrument to establish an automatic control system which will facilitate cell localization and encourage further research.

## 2. System Design

**Figure 1.**
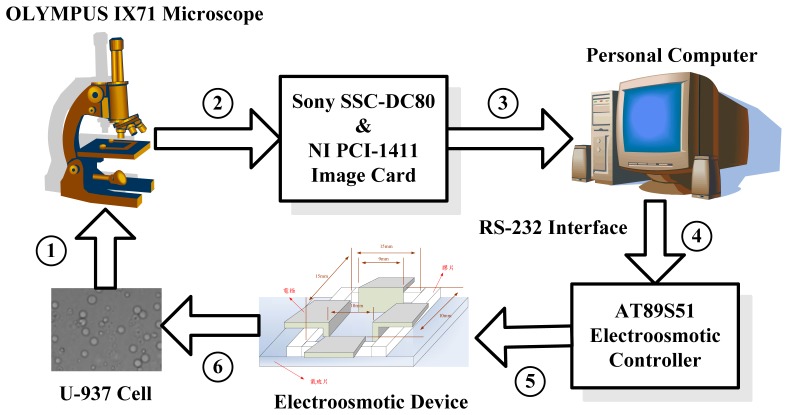
Schematic block in the present study.

The objective of the present study was to use machine vision to track a single cell using automatic electric-field strength and direction control, as well as an electroosmotic flow method to drive the two-dimensional free movement of the cell and allow localization at any position. To achieve this objective, the establishment of an image software system and a control hardware system is necessary. The graphical NI LabVIEW language was applied to create a human-machine interface for the operation of both machine vision and automatic control [9]. The system block is shown in Figure 1. The U-937 cell image observed under an OLYMPUS IX71 microscope is transmitted to the computer through an image acquisition card. After image processing, the control parameters are sent to the AT89S51 control circuit through an RS-232 interface. In this way, the two-dimensional movement of the U-937 cell is controlled. The software and hardware design is described below.

### 2.1. Image Software System

The image software system acquires a cell image which can be displayed on the computer, treats the acquired image further, tracks the coordinates and size of the controlled cell, and executes fuzzy operation for system control.

Image processing cannot take long given that real time control is required. The NI PCI-1411 image-acquisition card with a 640 × 480 gray scale resolution can acquire 30 images per second. A number of matrix operations may be applicable if image processing is used. Attaining the desired processing speed is impossible. To shorten the image processing time, fast operation methods must be adopted. However, such images will not be of good quality, and recognition and tracking may be affected to a certain degree. In principle, in determining whether processing time or image quality should be prioritized, the desirable answer is to have no delay conditions for the acquired image; having a lower failure rate of tracking is preferable.

#### 2.1.1. Image Processing

**Figure 2.**
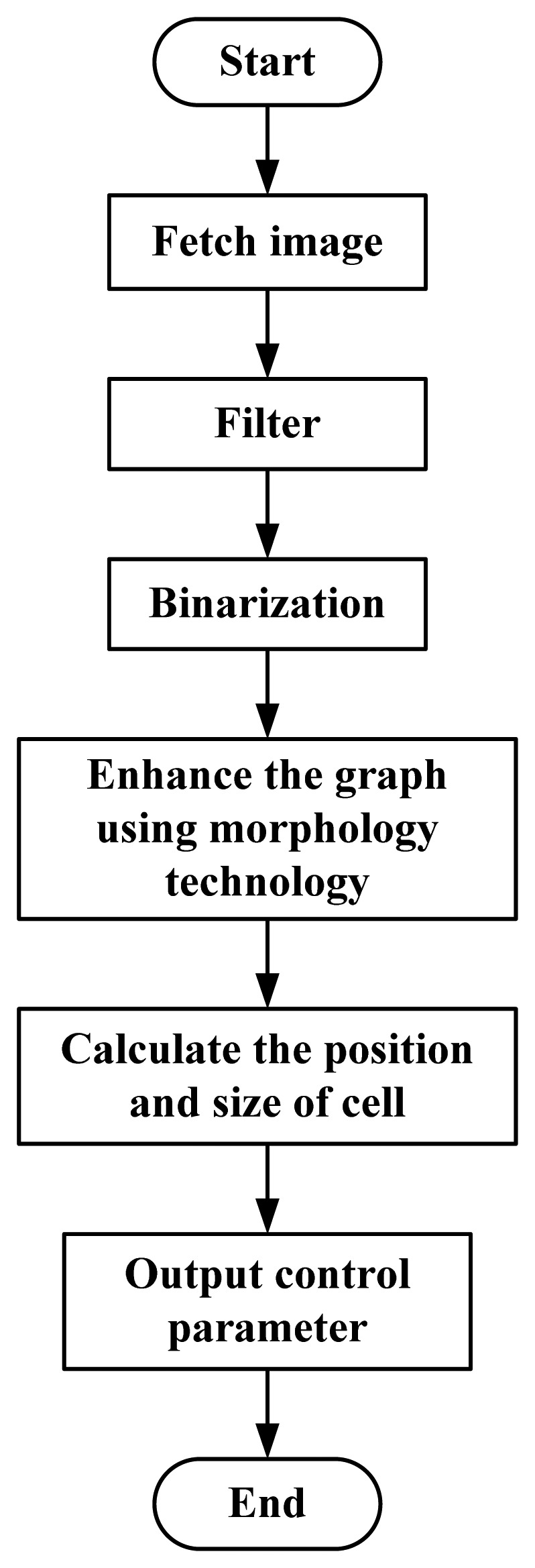
Flow chart of the image software system.

Because the acquired image will have noise, the image should be filtered to eliminate noise and obtain a smoother picture. In the beginning, an acquired image should be stored for observation, at this point the original image is no longer suitable for the image processing of cell tracking. In this experiment, binarization and morphological processing of the image were conducted to realize accurate cell manipulation [10]. Figure 2 represents the flow chart for the image processing software system.

**Figure 3.**
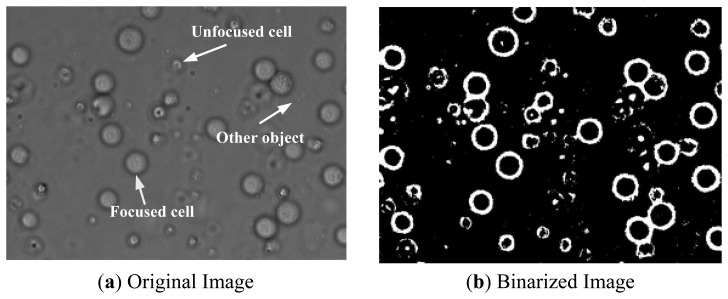
The compared picture after image binarization.

The NI Vision software by National Instruments has an IMAQ Local Threshold function for image binarization. It may use Background Correction or Ni black subVI to split the picture into different pieces before binarization. Bright Object or Dark Object subVI are used to seek relatively bright or dark areas. Background Correction methods were used for image binarization. The compared picture after image binarization is shown in Figure 3.

**Figure 4.**
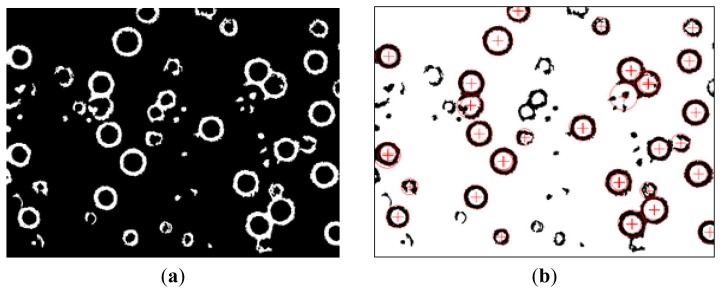
(**a**) The image after NI IMAQ Removed Particle, (**b**) The image after NI IMAQ Detected Shapes (black and white as opposite to original image).

From the binarized images, it can be seen that some doubtful, dead cells and other objects are retained in addition to the desired images. At this point IMAQ RemoveParticle subVI can be used to eliminate small impurities, clean up the images and prevent their influence on further detection (Figure 4(a)). Due to the fact U-937 cells have a circular configuration, they appear to have circular characteristics after preliminary image processing. Thus, by detecting the characteristic circular shape, the cell position coordinates and size can be determined. The IMAQ Detect Shapes subVI can find all approximate circles, and the location and size of every cell should be found quickly (Figure 4(b)).

**Figure 5.**
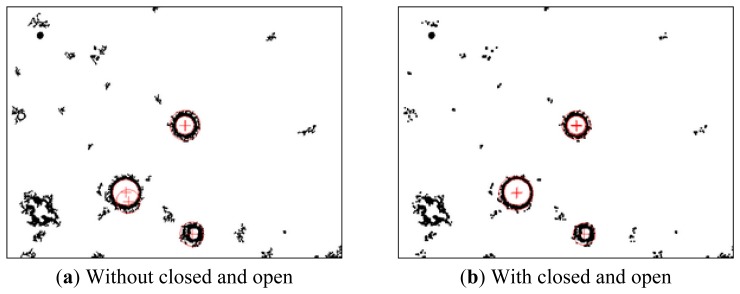
The compared image after closed and open image subVI operations.

The experiment also reveals that sometimes the retained ring thickness is uneven, or the outer ring is not uniform and has bumps. In such cases, the IMAQ Detect Shapes subVI may detect smaller, larger, or off-the-cell-center circles. Therefore, prior to detecting circles, image binarization processing for morphology is necessary. The binary image morphological algorithm is based on dilation or erosion operations. Gonzalez and Woods stated that dilation operations could enlarge or thicken the object, and erosion operations could shrink or thin it [6]. Using these two operations alternately could serve two functions: open and closed image functions. Open image subVI uses erosion first, followed by dilation, to separate bonded objects; close image subVI uses dilation first, followed by erosion, to bond separated objects. After practical experiments, the use of open and closed image subVIs from NI Vision software led to significant improvements in the bonding and separation of objects. Figure 5 indicates that the use of open and closed image subVIs can correct eccentric problems.

#### 2.1.2. Verifying Cell Location and Tracking

**Figure 6.**
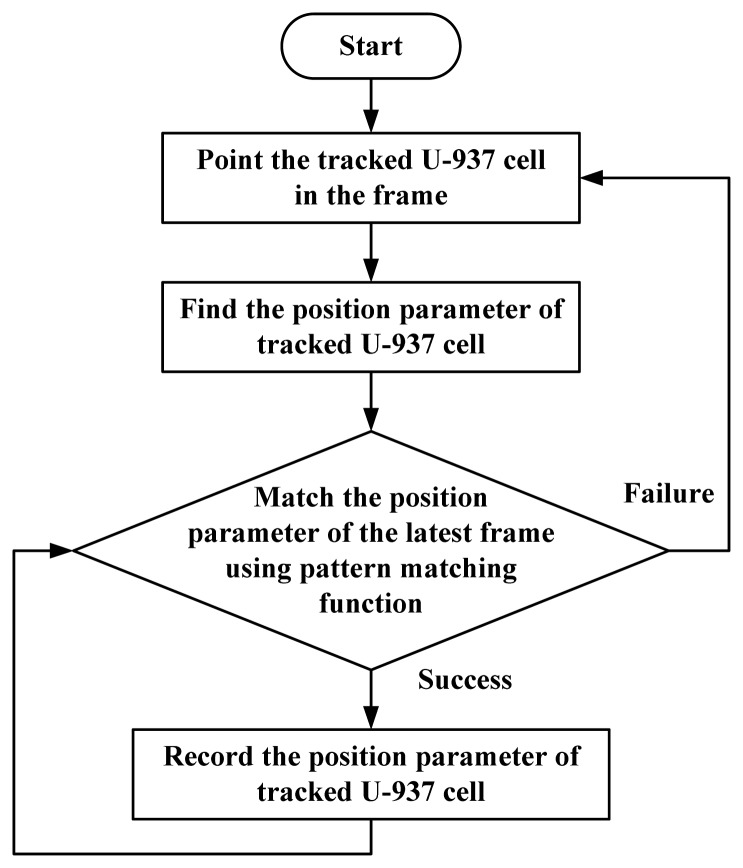
Flow chart for cell tracking.

After image processing, normal cells appear as regular circles; abnormal cells could be smaller, shrunk or deformed. A flow chart showing the tracking of cells is shown in Figure 6. At first, the PC mouse is moved to the tracked U-937 cell and clicked one time in the image frame to choose the target cell to be tracked. The system then compares the location coordinates of the mouse with respect to the location of all the circles in order to determine the target cell to be tracked and to complete the tracking behavior. The cell image is stored in the computer memory *via* a self-learn pattern function for the basis of identification, and the Pattern Matching subVI is then used to identify the cell in the next picture.

Pattern Matching subVI can only find a few of the most similar cells, but cannot identify the correct tracked cell. Every cell location sought by Pattern Matching is compared to the location recorded in the previous frame. It is known from the experiment that the speed of cell movement is not fast and its displacement does not exceed double the measured cell radius. Thus, the cell with the shortest distance which does not exceed double the radius is considered as the tracked cell in the frame of interest. Currently, this study is designed for tracking a single cell and cannot track more than one cell simultaneously.

### 2.2. Control Hardware System

**Figure 7.**
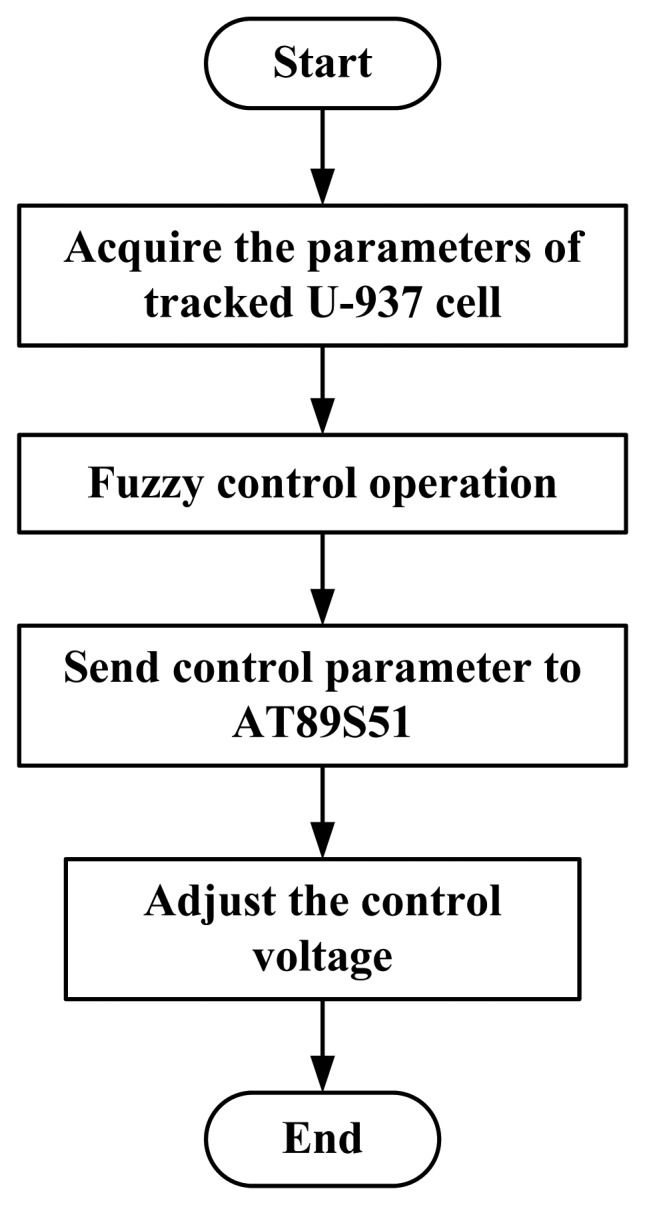
Flow chart of electroosmotic control.

The control hardware system changes the strength and direction of an electric field to control the movement of cells in a medium. The flow chart of electroosmotic control is shown in Figure 7. After image processing, the software system provides the parameters for the tracked single cell. Fuzzy logic is used to calculate the control parameters and transmit them through the RS-232 to the control circuit based on an AT89S51 single chip [11]. The AT89S51 single chip controls four relays to change the strength and direction of the control voltage in the actual hardware control.

#### 2.2.1. Electroosmotic Architecture

When the slide with negative surface charge *S_i_O*^−^ contacts an electrolyte medium, it is covered by many cations, which form an almost immobile stern layer. The excessive cations diffuse to form a diffuse layer. When the electric double layer is subjected to an external electric field, cations from the diffuse layer will move toward the negative electrode. This phenomenon is known as electroosmotic flow (EOF). Because EOF velocity is proportional to the electric field, changing the electric field intensity is sufficient to control the EOF velocity. Control of the strength and polarity of the electric field was implemented to change the speed and direction of EOF that can drive cell movement in the micro-buffer solution, thus achieving movement of the designated target cell toward its destination.

The EOF velocity is proportional to the electric field strength (*E*): this relationship is shown in Equation (1):

(1) $$u_{\text{eof}}=\mu_{\text{eof}}E$$

where *u*_eof_ is the EOF velocity (m s^−1^), and μ_eof_ is the EOF rate (m^2^ V^−1^ s^−1^). The electric field is the electric potential difference in unit distance. Given that the distance is fixed, a change in voltage (▽V); means a change in the electric field strength, namely:

(2) E∝∇V

(3) $$u_{\text{eof}}\propto \mu_{\text{eof}}\nabla V$$

**Figure 8.**
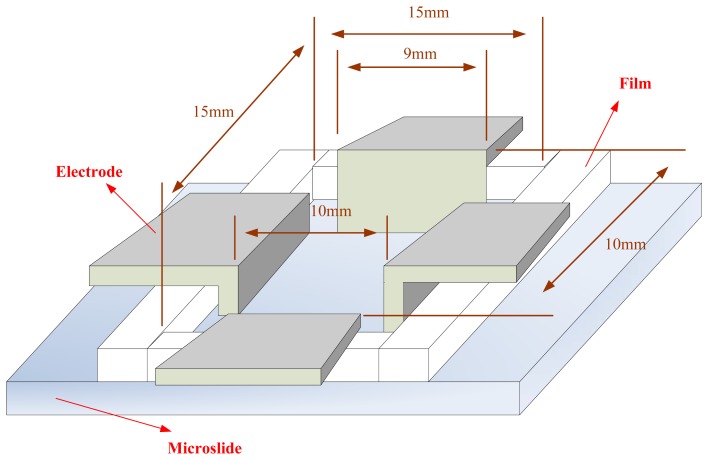
Schematic diagram of electroosmotic architecture.

The electroosmotic flow device for the current experiment involves cutting a 15 mm × 15 mm square from a plastic film and placing it onto a slide. The aluminum electrode is then placed around the four sides. This architecture is shown in Figure 8.

#### 2.2.2. Control Method

The PC mouse can be used to select a cell as the object to be controlled. In this study, the system automatically uses the target coordinates as the center, and uses the set value of the “Ratio” function on the control panel multiplied by the radius of the tracking cell as the localization radius in order to draw a circle as the target criterion. To change the target location, the user can use the mouse to click the desired place on the acquired image frame. After setting the tracked single cell and its target location, electrode direction is determined and voltage is calculated using fuzzy control theory. By definition, when the cell center is within the circular range, it is considered to be a successful localization.

**Figure 9.**
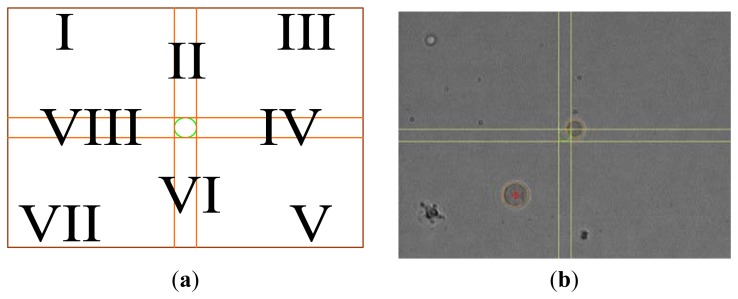
(**a**) The demarcated diagram of electric field direction (**b**) Real view on the PC screen; target radius ratio: 0.50.

The present study uses the target location as the center and divides the other acquired image frames into eight sections. The applied electric field direction depends on the section where the tracked cell is located, and is shown in Figure 9(a). I, III, V and VII are slanted voltages, whereas II, IV, VI and VIII are horizontal or vertical direction voltages. The direction of the electric field runs from high to low electric potential. Hence, two output terminals can have different voltages within the circuit design. If only a single-direction electric field is applied, the electrode in the other direction is floating. As in Figure 9(b), the green circle in an actual experiment becomes the target circular range. The cell with the red label is the tracked cell, and the yellow line is the demarcated reference for applied electric field direction.

We expect the movement of cells to be steady and fast. They should move fast when the distance from the localized point is considerable and approach slowly when the distance is short. Because the EOF is proportional to the electric field, the flow speed can be controlled by changing the intensity of the electric field. For the control rule, fuzzy logic was used to calculate the optimal relationship between the distance and applied voltage. Usually, fuzzy logic theory is developed to help solve challenging real world problems. It was first proposed by Zadeh introduced the concept of linguistic variables, which in this article equates to a variable defined as a fuzzy set [12]. Fuzzy control is a practical alternative for a variety of challenging control applications.

**Figure 10.**
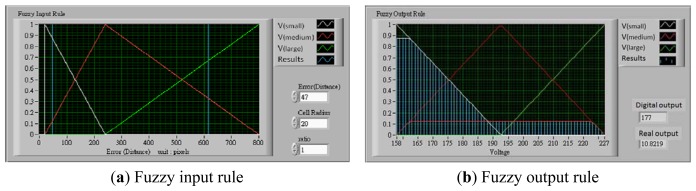
The fuzzy output rule in this control platform.

The voltage controller is developed based on the center of gravity defuzzification. Given that the size of the acquisition image frame is 640 × 480 pixels in length and width, its diagonal length is 800 pixels. The largest movement distance is 800 pixels when the tracked cell and target location are located at the end point of the diagonal, and the smallest movement distance is the radius of the tracked cell. The movement distance changes with cell size and the radius ratio setting. The fuzzy membership functions are:

(4.a) $$\mu_{\left(\text{small}\right)}\;(x)=\frac{-(x-240)}{240-(\text{Radius x radio})},(\text{Radius x radio})\leq x\leq 240$$

(4.b) $$\mu_{\left(\text{medium}\right)}\;(x)=\frac{\left(x-\text{Radius}\right)}{240-\text{Radius}},\text{Radius}\leq x\leq 240$$

(4.c) $$\mu_{\left(\text{medium}\right)}\;(x)=\frac{-(x-800)}{800-240},240\leq x\leq 800$$

(4.d) $$\mu_{\left(\text{large}\right)}\;(x)=\frac{\left(x-240\right)}{800-240},240\leq x\leq 800$$

where *Radius* is the radius of the tracked cell, and *ratio* is a scale parameter. The value μ(*x*) is called the membership degree of *x* in the fuzzy set. The membership degree μ(*x*) quantifies the grade of membership of the element *x* to the fuzzy set. The value 0 indicates that *x* is not a member of the fuzzy set; the value 1 means that *x* is a full member of the fuzzy set. The movement distance data were processed and transferred to output control voltage by the triangle center of gravity defuzzification [13,14]. The fuzzy output rule is shown in Figure 10. For example, if the voltage range is set as 9.2–19.76 V, when the movement distance between the tracked cell and the target location is 47 pixels (short distance), the radius is 20 and the ratio is 1, see Figure 10(a). The value of μ_(small)_(*x*) is 0.8772 and μ_(medium)_(*x*) is 0.1227. The digital output (*D_o_*) is 177 in accordance with the triangle center of gravity defuzzification. The output voltage (*V_o_*) is relayed to the *D_o_*, as shown in Equation (5). The real *V_o_* is 10.82 volts, as shown in Figure 10(b).

(5) $$V_{0}=0.2357\;x\;D_{o}-30.897$$

#### 2.2.3. Control Circuit Design

**Figure 11.**
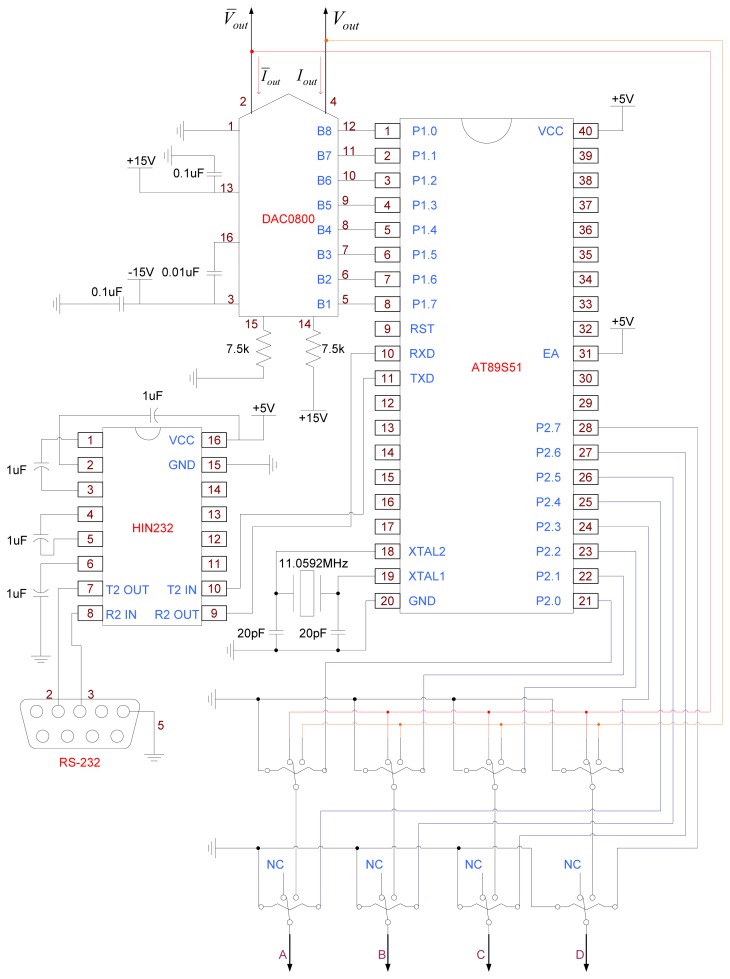
Control circuit diagram.

As shown in Figure 11, the control circuit is based on an AT89S51 single chip and uses RS-232 as interface for communication with a computer to receive two control data. First, four relays are used to control A, B, C and D electrodes for changing the electric field direction. In addition, an 8 bit DAC0800 IC is used to convert digital signals into an analog output voltage (*V_out_*, ^∼^*V_out_*).

## 3. Experimental Equipment and Methods

The experimental equipment and method are as follows:

### 3.1. Experimental Equipment

The tested cells consist of human histiocytic lymphoma U-937 obtained from the Food Industry Research and Development Institute (Hsinchu, Taiwan). Table 1 contains the basic information for the U-937 cells. The experimental equipment is listed in Table 2. A reverse microscope, microscopy camera, image acquired card and self-made electroosmotic flow control circuit are among the equipment employed. NI LabVIEW 8.2 and NI Vision 8.2 software were used also.

### 3.2. Experimental Method

The objective of the experiment was to control the cell by automatic positioning. Before the experiment, the cell image was recorded for testing and facilitating parameter settings by moving the microscope platform to change the cell location on the screen. An image of good quality helps further image processing. The experiment procedures were as follows:

(1) The cell solution was centrifuged, after which the supernatant was removed. Up to 0.1 M of aqueous glucose solution was added and mixed well with the cell solution.
(2) Electroosmotic devices were installed and wires connected to the electrodes.
(3) Approximately (200 to 300) mL of 0.1 M aqueous glucose solution was added to 10 μL of cell solution.
(4) The system program was run with automatic control functions and a graphical human machine interface.

Most parameters can be automatically set when running the image system program. Only the parameter settings of brightness and darkness depend on environmental conditions. The control hardware system parameters can be adjusted at any time. The control circuit can start after all of the parameters have been set.

After system testing, it was found that the time of voltage supply was different in slightly different calculations. Although the difference was only a few milliseconds, this could cause instability in relay control circuit. To increase efficiency and reduce any instability, a time factor was added as a control factor. The generated function for time factor is the same as that for the voltage factor. These are determined by the fuzzy logic control method described earlier. What follows discusses the effect of voltage, time and the combination of voltage and time as three control factors.

### 3.3. Voltage as a Control Factor

**Figure 12.**
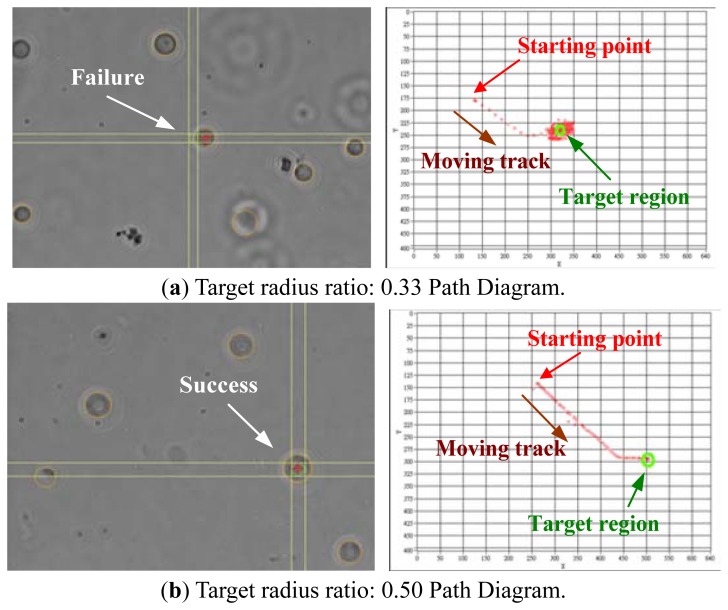
(**a**) A real failure diagram as the target radius ratio is too small; (**b**) A real successful diagram as the target radius ratio is appropriate.

During the experiment, the control parameters were set first. A single cell was selected randomly and moved to any place on the image screen. Success was dependant on the target radius ratio. As seen in Figure 12(a), if the target circular range was too small, the tracked cell moved around the target circular range and could not stop within the circular target range to enable us to find a suitable target radius ratio parameter for successful positioning. If the microscope magnification is different, it is necessary to find an appropriate target radius ratio before beginning the experiment.

The path diagram in Figure 12(b) indicates if the cell is positioned directly. There are no multiple deflections or return traces. Increasing the radius ratio can obtain good results. It can be determined from Table 3 that when the target radius is increased to match the cell radius, the final result is an almost center positioning by the planned path with a successful rate up to 100% (*i.e.*, the *Error (Distance)* value is less than the *Cell Radius x ratio*, as shown in Equation (6)). Therefore, different voltage settings are used to change movement velocity. The ideal path and shortest positioning time occur when the microscope magnification is 20X (10 μm = 20.22 pixel) and the target radius ratio is 1.0:

(6) Error(Distance)≤Cell Radius x ratio

### 3.4. Time as a Control Factor

Although the success rate can reach 100% with voltage as the control factor, the positioning precision is low because the cell movement is too fast to stop when it approaches the target location. With lower driving voltage, the situation does not improve much. If the time of voltage supply is decreased, the movement velocity can also be changed. Therefore, the time of a constant voltage supply is adopted as the control factor. When the cell is far from the positioning target, the voltage application time is long; when the distance is near, the time is short.

Following experiments with voltage as control factor, the suitable parameter for successful positioning was determined. After repeating the above voltage factor experiments, when the microscope magnification is 40X (10 μm = 40.44 pixel), the radius ratio is 0.2 and the output voltage is 19.2 volt, a 100% successful rate can be obtained for positioning.

### 3.5. Combination of Voltage and Time as a Control Factor

The advantages of using voltage as a control factor are high speed and shorter positioning time, while the advantage with time as a control factor is high precision. Here, these two control factors are combined. If the distance between the tracked cell and target location is larger than the target circular range, the driving mode is executed by voltage; if the distance is smaller than the target circular range, the driving mode is executed by time. Experimental results were observed to determine the characteristics of fast positioning and high precision.

## 4. Results and Discussion

### 4.1. Results Using Voltage as Control Factor

**Figure 13.**
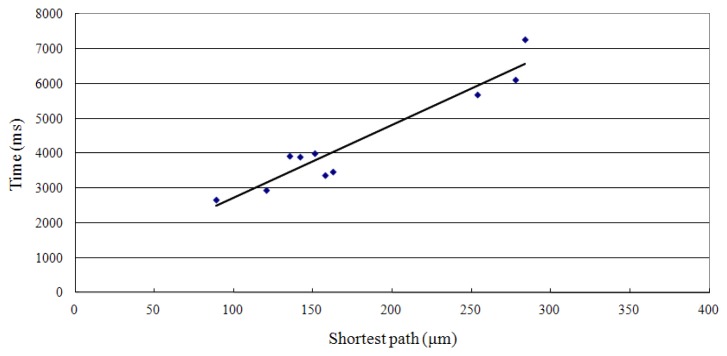
Relationship between the positioning time and the shortest path with voltage as the control factor.

When the microscope magnification is 20X (10 μm = 20.22 pixels), and the target radius ratio is 1.0, the positioning success rate by voltage control can reach 100%. Table 4 shows 10 sets of data using voltage as a control factor. The shortest path is the straight line between the starting point coordinate and the target region coordinate. The real path is the cell moving track during positioning. The velocity value in Table 4 is defined as the shortest path divided by time, whereas the speed value is defined as the real path divided by time. Figure 13 demonstrates the relationship between the shortest path and the positioning time.

From the trend indicated in Figure 13 (Y = 20.929X + 618.75, R^2^ = 0.9149), the longest distance is estimated to be 197.82 μm (when the microscope magnification is 20X, half the diagonal line is 400 pixels, so that the distance is about 197.82 μm). The required positioning time is about 4,759 ms (20.929 × 197.82 + 618.75 = 4759, within 5 s).

### 4.2. Results Using Time as Control Factor

When the microscope magnification is 40X (10 μm = 40.44 pixels) and the target radius ratio is less than 0.2, the positioning success rate by time control can reach 100%. Table 5 shows 10 sets of data using positioning time as a control factor.

**Figure 14.**
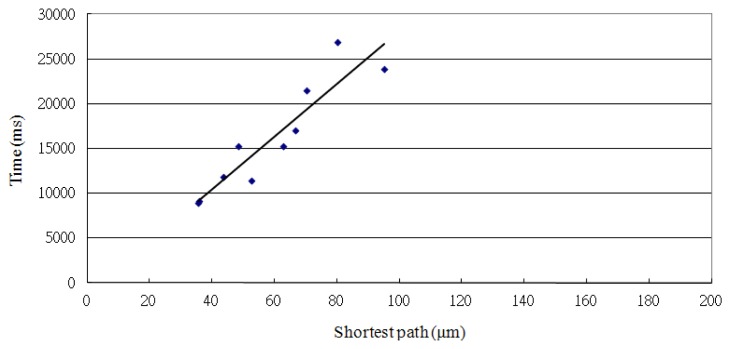
Relationship between the positioning time and the shortest path with time as the control factor.

Based upon the trend displayed in Figure 14 (Y = 293.66X–1269.4, R^2^ = 0.8492), the longest distance is estimated to be 98.91 μm, and the required positioning time is 27,777 ms (293.66 × 98.91–1269.4 = 27777) within 28 s. Compared with voltage as the control factor, the time control method is more precise and accurate but takes about 5.6 times longer than the voltage method to complete.

### 4.3. Results Using a Combination of Voltage and Time as Control Factor

**Figure 15.**
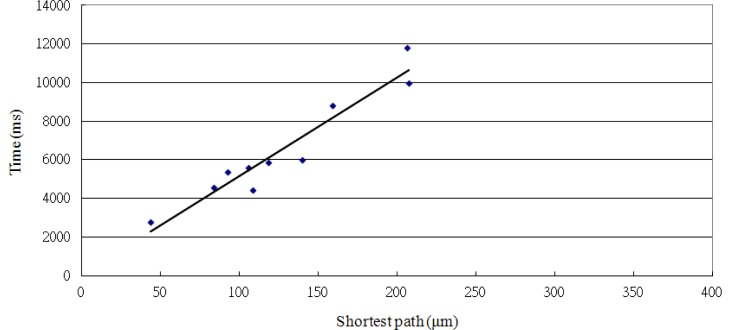
Relationship between the positioning time and the shortest path with a combination of voltage and time as the control factor.

When the microscope magnification is 20X and target radius ratio is less than 0.2, the positioning success rate by both voltage and time control can reach 100%. Table 6 also shows 10 sets of data using a combination of voltage and time as the control factor. Based on the trend indicated in Figure 15 (Y = 50.97X + 51.18, R^2^ = 0.9157), the longest distance is 197.82 μm and the required positioning time is 10,134 ms (50.97 × 197.82 = 10,134). Compared to the voltage control method or the time control method alone, the combined method is more precise and accurate than the voltage method, and faster than the time method. It has advantages in high speed and enhanced precision and accuracy. Its control speed is about 5.18 times (19.523/3.77 = 5.18) greater than the time method and the control precision is about 5 times (1.0/0.2 = 5) more accurate than the voltage method. Table 7 shows the relationship between the different control factors and the speed or velocity. Speed and velocity are as shown in Equations (7) and (8), respectively:

(7) $$\text{Speed}=\frac{\text{Shortest Path}}{\text{Time}}$$

(8) $$\text{Velocity}=\frac{\text{Real Path}}{\text{Time}}\#$$

## 5. Conclusions

This study combined the electroosmotic flow, image processing, and automatic control technologies for biomolecular manipulation. The results may be applied to preparatory procedures for single-cell research. For example, when a laser is adopted, given that changing the laser incident point is difficult, the cell is usually moved to the incident point. The system developed can provide fast and accurate cell movement to the laser incident point and achieve automatic positioning. The results can be combined with other microelectromechanical devices to integrate other systems into one microfluidic chip as a micro total analysis system.

The results of the current experiments indicate that the success rate of the voltage control method can reach 100% when the target radius ratio is 1.0 and positioning is completed within 5 s. The success rate of the time control method can also reach 100% when microscopic magnification is increased to 40X, the target radius ratio is 0.2, and positioning is completed within 28 s. The success rate of the combined voltage and time control method can also reach 100% when the microscopic magnification is increased to 20X and the target radius ratio is less 0.2. The control speed using the combined voltage and time control method is about 5.18 times greater than that achieved using the time control method, and the control precision is more than five times more accurate than the voltage method.

## Figures and Tables

**Table 1.** Cell information.

**Source**	**Human Histiocytic Lymphoma**
Name	U-937
Origin	ATCC CRL-1593.2
Characteristic	Suspension
Medium	90% RPMI 1640 medium with 2 mM L-glutamine adjusted to contain 1.5 g/L sodium bicarbonate, 4.5 glucose, 10 mM HEPES, 1.0 mM sodium pyruvate + 10% fetal bovine serun.
Condition	37 °C, 5% CO_2_

Source: Food Industry Research and Development Institute in Taiwan.

**Table 2.** Software and hardware.

**Device Name**	**Type**	**Purpose**
Inverted Microscope	OLYMPUS IX71	View Sample
Camera, Image Card	Sony SSC-DC80, NI PCI-1411	Fetch Image
Graphical Software	NI LabVIEW 8.2, NI Vision 8.2	System Interface, Image Processing, Fuzzy Control
Electroosmotic Controller	DIY	Adjust the Control Voltage

**Table 3.** The relationship between radius ratios and positioning successful rate with voltage as control factor.

**Radius Ratio**	**Magnification**	**Successful Rate**
0.333	20X (10 μm = 20.22 pixel)	14.29%
0.5	20X (10 μm = 20.22 pixel)	83.34%
1	20X (10 μm = 20.22 pixel)	100.0%

**Table 4.** Experiment data with voltage as control factor.

	**Shortest Path (μm)**	**Real Path (μm)**	**Time (ms)**	**Speed (μm s^−1^)**	**Velocity (μm s^−1^)**
1	162.654	177.505	3,474	46.82	51.10
2	89.210	104.654	2,672	33.39	39.17
3	135.552	158.292	3,933	34.47	40.25
4	253.817	264.855	5,674	44.73	46.68
5	277.534	288.793	6,101	45.49	47.34
6	283.955	301.384	7,263	39.10	41.50
7	120.713	141.255	2,933	41.16	48.16
8	141.705	155.091	3,898	36.35	39.79
9	150.909	174.825	4,002	37.71	43.68
10	157.718	165.578	3,361	46.93	49.26
Average	177.38	193.22	4,331.10	40.61	44.69

**Table 5.** Experiment data with time as control factor.

	**Shortest Path (μm)**	**Real Path (μm)**	**Time (ms)**	**Speed (μm s^−1^)**	**Velocity (μm s^−1^)**
1	70.405	86.237	21,528	3.27	4.01
2	35.968	37.056	9,086	3.96	4.08
3	35.565	42.764	8,879	4.01	4.82
4	52.762	62.661	11,446	4.61	5.47
5	95.24	112.659	23,846	3.99	4.72
6	80.276	132.587	26,901	2.98	4.93
7	62.912	64.125	15,265	4.12	4.20
8	48.368	52.149	15,264	3.17	3.42
9	43.587	75.259	11,871	3.67	6.34
10	66.632	104.094	16,983	3.92	6.13
Average	59.17	76.96	16,106.90	3.77	4.81

**Table 6.** Experiment data with both voltage and time as control factor.

	**Shortest Path (μm)**	**Real Path (μm)**	**Time (ms)**	**Speed (μm s^−1^)**	**Velocity (μm s^−1^)**
1	44.06	53.17	2,781	15.84	19.12
2	108.71	113.53	4,426	24.56	25.65
3	84.12	95.08	4,574	18.39	20.79
4	206.36	235.22	11,813	17.47	19.91
5	92.64	109.64	5,358	17.29	20.46
6	207.75	255.77	9,973	20.83	25.65
7	159.53	172.11	8,811	18.11	19.53
8	105.91	118.77	5,574	19.00	21.31
9	118.61	126.79	5,839	20.31	21.71
10	140.04	143.96	5,978	23.43	24.08
Average	126.77	142.40	6,512.70	19.52	21.82

**Table 7.** Relationship between the different control factors and the speed or velocity.

**Speed or Velocity**	**Speed (μm s^−1^)**		**Velocity (μm s^−1^)**	

**Control Factor**	**Mean**	**Std**	**Mean**	**Std**

Voltage	40.615	5.145	44.693	4.351
Time	3.770	0.499	4.812	0.944
Voltage & Time	19.523	2.775	21.821	2.445
